# A lightweight network for improving wheat ears detection and counting based on YOLOv5s

**DOI:** 10.3389/fpls.2023.1289726

**Published:** 2023-12-18

**Authors:** Xiaojun Shen, Chu Zhang, Kai Liu, Wenjie Mao, Cheng Zhou, Lili Yao

**Affiliations:** School of Information Engineering, Huzhou University, Huzhou, China

**Keywords:** wheat ears counting, lightweight, YOLOv5S, ShuffleNetV2, DyHead

## Abstract

**Introduction:**

Recognizing wheat ears plays a crucial role in predicting wheat yield. Employing deep learning methods for wheat ears identification is the mainstream method in current research and applications. However, such methods still face challenges, such as high computational parameter volume, large model weights, and slow processing speeds, making it difficult to apply them for real-time identification tasks on limited hardware resources in the wheat field. Therefore, exploring lightweight wheat ears detection methods for real-time recognition holds significant importance.

**Methods:**

This study proposes a lightweight method for detecting and counting wheat ears based on YOLOv5s. It utilizes the ShuffleNetV2 lightweight convolutional neural network to optimize the YOLOv5s model by reducing the number of parameters and simplifying the complexity of the calculation processes. In addition, a lightweight upsampling operator content-aware reassembly of features is introduced in the feature pyramid structure to eliminate the impact of the lightweight process on the model detection performance. This approach aims to improve the spatial resolution of the feature images, enhance the effectiveness of the perceptual field, and reduce information loss. Finally, by introducing the dynamic target detection head, the shape of the detection head and the feature extraction strategy can be dynamically adjusted, and the detection accuracy can be improved when encountering wheat ears with large-scale changes, diverse shapes, or significant orientation variations.

**Results and discussion:**

This study uses the global wheat head detection dataset and incorporates the local experimental dataset to improve the robustness and generalization of the proposed model. The weight, FLOPs and mAP of this model are 2.9 MB, 2.5 * 10^9^ and 94.8%, respectively. The linear fitting determination coefficients R^2^ for the model test result and actual value of global wheat head detection dataset and local experimental Site are 0.94 and 0.97, respectively. The improved lightweight model can better meet the requirements of precision wheat ears counting and play an important role in embedded systems, mobile devices, or other hardware systems with limited computing resources.

## 1 Introduction

Wheat is one of the primary food crops, and its steady yield is required for both economic growth and human food security (Xiao et al., 2020). Wheat ears counting is an important method to predict wheat yield. However, conventional methods often rely on subjective and time-consuming visual inspection, making it difficult to identify and count wheat ears rapidly and accurately (Chen et al., 2017; Fernandez-Gallego et al., 2020). With the progression of image recognition technology, utilizing image data for wheat ears detection and counting has become a more effective approach. (Prakash et al., 2017). Traditional image processing methods can achieve wheat ears counting in simple environments, but they also have shortcomings such as complexities in feature engineering, low recognition accuracy, and weak transferability. Relatively, the deep learning-based method for wheat ears recognition provides a more efficient practical solution. However, higher accuracy also entails an increased number of parameters and large computational costs. These factors restrict the lightweight application of these methods, preventing their deployment on mobile devices for the swift execution of recognition tasks.

In recent years, most deep learning-based wheat ears recognition technology have employed various methods to optimize the backbone network, feature pyramid module, and loss function of the existing models. Although multiple lightweight wheat ears detection models have been proposed, the deployment and operation of these lightweight models still face significant challenges on embedded systems and mobile devices. To address this issue, it is essential to conduct in-depth research into a lightweight real-time wheat ears detection model that is better suited for devices with lower computational capabilities and limited memory in embedded systems and mobile devices.

This paper proposes a lightweight wheat ears detection method, S-YOLOv5s, based on YOLOv5s. By optimizing the backbone network, neck network, and head network of the model, the weight, parameters, and computational load of model are significantly superior to previous methods. The model can be applied to real-time detection tasks on low-performance devices in complex field environments. The main contributions are summarized as follows:

- Establishing a completely new wheat ears dataset by incorporating the local experimental site into the Global Wheat Head Detection Dataset (GWHD) to enhance the robustness and generalization of the proposed model.
- Reducing the parameters and computational complexity of the proposed model by adopting SmallConv (3 x 3 Conv) and the lightweight network ShuffleNetV2 to replace the backbone network (CSPDarknet) of the YOLOv5s model.
- Introducing the lightweight upsampling operator Content-Aware ReAssembly of Features (CARAFE) to replace the upsampling operation in the Path Aggregation Network (PANet). It improves feature information extraction, enhances the spatial resolution of feature maps, and addresses challenges in detecting difficult scenarios such as high wheat density and severe occlusion.
- Utilizing the attention-based Dynamic Target Detection Head (DyHead) to flexibly adjust the shape of the detection head and feature extraction strategy, enabling adaptation to the diversity of wheat ears in different scales, shapes, and orientations.

## 2 Literature review

The current methods for wheat ears recognition mainly include conventional image recognition and deep learning-based techniques (Yuan et al., 2022). Traditional image recognition techniques typically use feature extraction algorithms and image processing techniques to obtain wheat ears features (such as filtering, edge detection, etc.) in order to accurate identify wheat ears (Grillo et al., 2017; Dhingra et al., 2018; Ganeva et al., 2022). The frequently used features include edge, corner point, texture, temperature, color histogram, etc. (Li et al., 2017; Yuan et al., 2020; Rui et al., 2022a; Dandrifosse et al., 2022). Fernandez-Gallego et al. (2019) used a thermal infrared camera to segment wheat ears based on the temperature difference between the wheat leaves and the wheat ears. This method can alleviate the effect of overlapping wheat ears on counting. However, obtaining useful photos becomes challenging when the temperature difference between wheat ears and leaves is minimal. Cheng et al. (2018) used multi-feature fusion (for example, color, texture, and special image features) and dual support vector machine methods (Gholami and Fakhari, 2017), efficiently achieving wheat ear counting by classifying pixels and performing image segmentation.

With the rapid development of deep learning technology, the capability of target detection models in image recognition and classification has significantly improved (Mu and Zeng, 2019). The target detection models are divided into single-stage models (e.g., Single Shot MultiBox Detector, You Only Look Once) and two-stage models (e.g., R-CNN, Faster R-CNN)(Ning et al., 2017; Bharati and Pramanik, 2020; Wu et al., 2022; Xu et al., 2023). Both the single-stage and two-stage models have high accuracy on wheat ears detection tasks. Mehedi et al. (2018) used a two-stage R-CNN model trained on wheat at various growth stages to generate four models, which can be used to classify the four growth stages of the spike dataset, the average detection accuracy of the model ranges between 88% and 94%. Compared to the two-stage model, the single-stage model YOLO has faster detection speed (Jing et al., 2021), fewer model parameters, and lower weight, which is more suitable for field lightweight applications, such as being deployed on mobile platforms for real-time detection tasks. Bhagat et al. (2021) proposed a lightweight wheat ears detection method called WheatNetLite based on YOLOv3. This model utilizes Mixed Depthwise Convolution (MDWConv) with inverted residual blocks to construct the backbone feature extraction network of WheatNetLite, reducing the parameter count of the model. As a result, the weight of the YOLOv3 model was reduced from 54.2 MB to 8.2 MB, while achieving a mean Average Precision (mAP) of 91.3%. Shi et al. (2023) constructed a dataset covering three growth stages of wheat: flowering, grain filling, and maturity. After performing pruning on the YOLOv5s algorithm, they proposed a new wheat ear detection algorithm named YOLOv5s-t by altering the convolution kernel sizes in the spatial pyramid to reduce the number of model convolutions, thereby decreasing the parameter count of the model. The model weight is 9.1 MB, indicating a reduction of 5.3 MB. The mAP is 97.4, reflecting a decrease of 1.09%. Shu et al. (2022) optimized the original YOLOv5 model structure using a local wheat dataset to optimize the structure of YOLOv5 using the GhostNet lightweight modules (Han et al., 2020), reducing the parameters and computational complexity of the model. They further transformed the loss function from Complete Intersection-Over-Union (CIOU) to Efficient Intersection Over Union (EIOU), directly minimizing the difference in width and height between target and anchor boxes (Zhang et al., 2021), which accelerated the convergence speed. The improved model boasts a mAP of 96.60%, a detection time of 0.0181s, and a model size of 8.12 MB, which is 2.3 MB smaller than YOLOv5.

In general, the target detection models based on deep learning demonstrate more robust functionality in object recognition tasks and are in line with the development trends of modern smart agriculture. Therefore, achieving model lightweighting based on existing research and applying it to small mobile devices is of significant importance.

## 3 Materials and methods

### 3.1 Data sources

**Figure 1:**
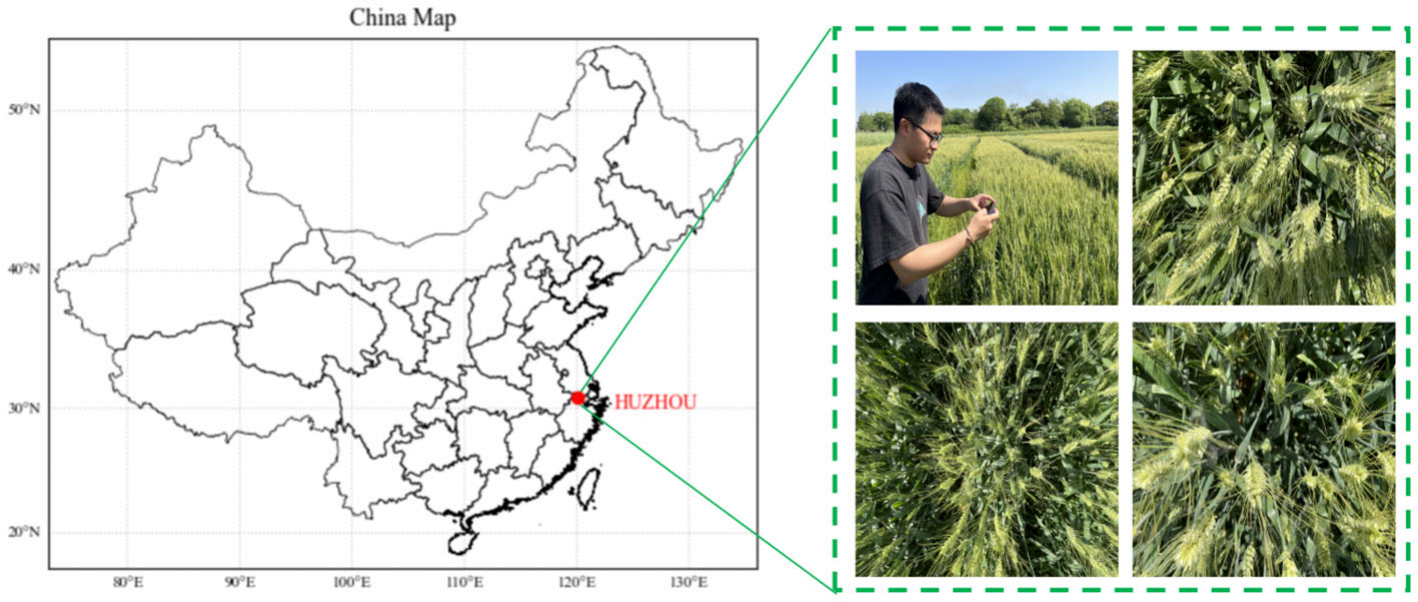
Experimental site and cell phone images of wheat ears.

Two different datasets were used in this study to ensure the accuracy and generalizability of the model training results, as outlined in **Table 1**. The first dataset (GWHD, http://www.global-wheat.com/) was sourced from the global wheat public dataset, from which 2090 images captured in complex scenes, including blurred, dark, and sunny conditions, were selected. An actual field experiment produced the second dataset (Experimental Site) in Huzhou City, Zhejiang Province, China, as shown in **Figure 1**. Under sunny conditions, wheat images at the heading stage were captured from multiple angles using an iPhone 12. The camera resolution is 3024 x 4032 pixels, and the camera was positioned approximately 400 - 600 mm above the wheat heads. A total of 527 images were saved in JPG format. Subsequently, image processing techniques were employed to introduce noise and reduce brightness to a subset of these images. Following this, the photos were scaled to 2048 x 2048 pixels through a resampling process to ensure that the image parameters met the requirements for model training.

**Table 1:** Wheat ears datasets.

Datasets	Image resolution	Blurred	Dark	Sunny	Training Set	Valid Set	Test Set	Images	Number of labels
GWHD	1024 x1024	366	74	1650	1672	209	209	2090	14876
Experimental Site	2048 x 2048	175	175	177	423	52	52	527	92184
Total		541	249	1827	2095	261	261	2617	107060

**Figure 2:**
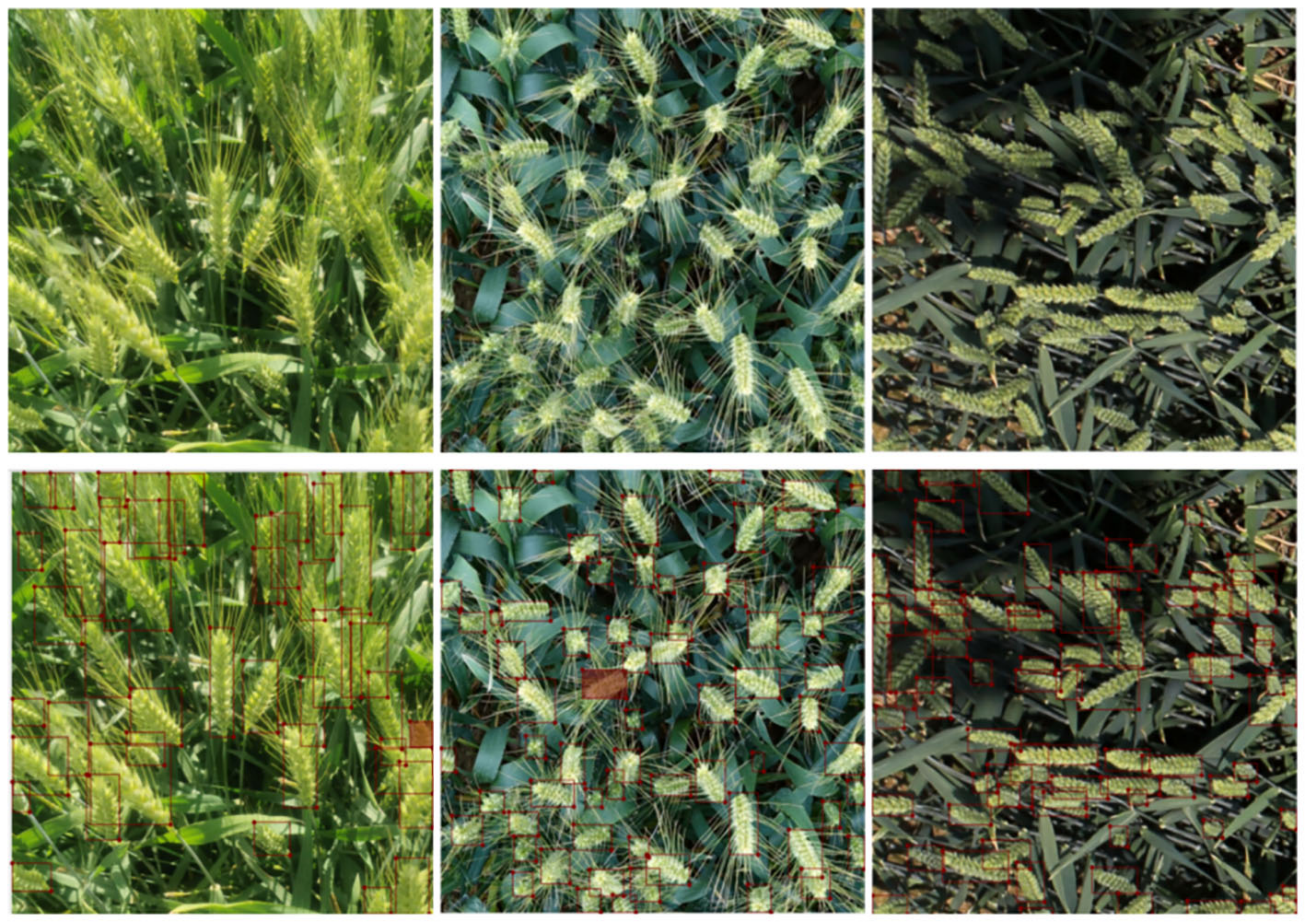
Manual annotation of images for datasets.

This study uses the machine learning and deep learning general image annotation tool labelme (http://labelme.csail.mit.edu/Release3.0/) to annotate the wheat images in the datasets. Once the labeling process is completed, a JSON data file containing the coordinate information and class names of the wheat ears is generated. Subsequently, this data is converted to a txt format using Python and serves as the training input of the model, containing 107,600 labels. The processed 2617 images were randomly divided into training set, validation set and test set in the ratio of 8:1:1, and the labeled images are shown in **Figure 2**.

### 3.2 Model structure and optimization

#### 3.2.1 YOLO model series

YOLO is a widely utilized single-stage object detection algorithm, and YOLOv5 represents the fifth iteration in this series. Compared to YOLOv7, YOLOv5 offers significant advantages in terms of inference speed, lightweight model weights, reduced memory usage, and rapid deployment. In comparison to YOLOv8, YOLOv5 is more hardware-friendly in deployment and exhibits superior frame rate performance on CPUs. This makes YOLOv5 a more efficient and practical choice, especially for applications that require lightweight object detection solutions. As a result, it is better suited to serve as a baseline for lightweight model research, and can perform real-time object detection in images. YOLOv5 is primarily divided into four versions: YOLOv5s, YOLOv5m, YOLOv5l, and YOLOv5x. This study primarily focuses on model optimization based on YOLOv5s. In comparison to other versions of the YOLOv5 algorithm, this model has fewer parameters and requires less computational resources. Additionally, three modules have been introduced in the YOLOv5s model: ShuffleNetV2, CARAFE, and DyHead.

#### 3.2.2 ShuffleNetV2

**Figure 3:**
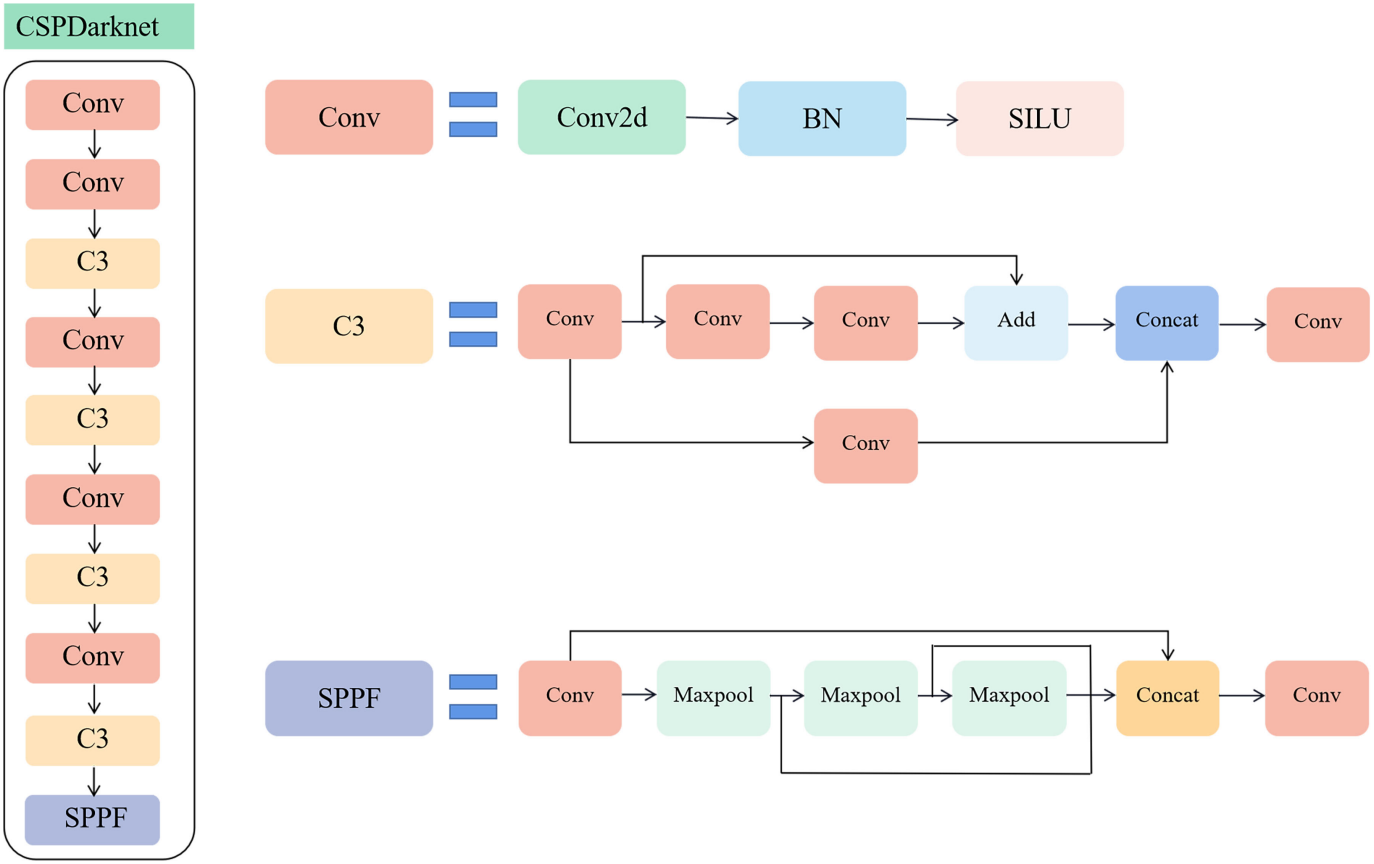
Network structure of CSPResNet50, the backbone network of YOLOv5s.

For the backbone network of the YOLOv5s model, CSPDarknet, as shown in **Figure 3**, the excessive use of convolutional layers and numerous cross-convolution operations lead to a significant amount of gradient information being reused for weight updates. The number of parameters and computing effort of the model both increased.

Therefore, this study attempts to replace the backbone feature extraction network of the YOLOv5s model with three currently popular lightweight networks: Ghost, MobileNetV3, and ShuffleNetV2 to reduce the parameter and computational complexity of the model. Comparative results are shown in **Table 2**, where ShuffleNetV2 achieves a significant advantage in lightweight network comparison with a model weight of 2.1 MB. Ghost and MobileNetV3, with model weights nearly 5 times and 3.5 times that of ShuffleNetV2, respectively, only outperform detection accuracy of ShuffleNetV2 by 2.6% and 1.2%. Therefore, this study adopts the SmallConv (3 x 3 Conv) and ShuffleNetV2 to replace the backbone feature extraction network of the YOLOv5s.

**Table 2:** Comparison results between the YOLOv5s algorithm and the fusion of three lightweight networks.

Model	Weight (MB)	mAP (%)
YOLOv5s + Ghost	10.2	95.5
YOLOv5s + MobileNetV3	7.16	94.1
YOLOv5s + ShuffleNetV2	2.1	92.9

**Figure 4:**
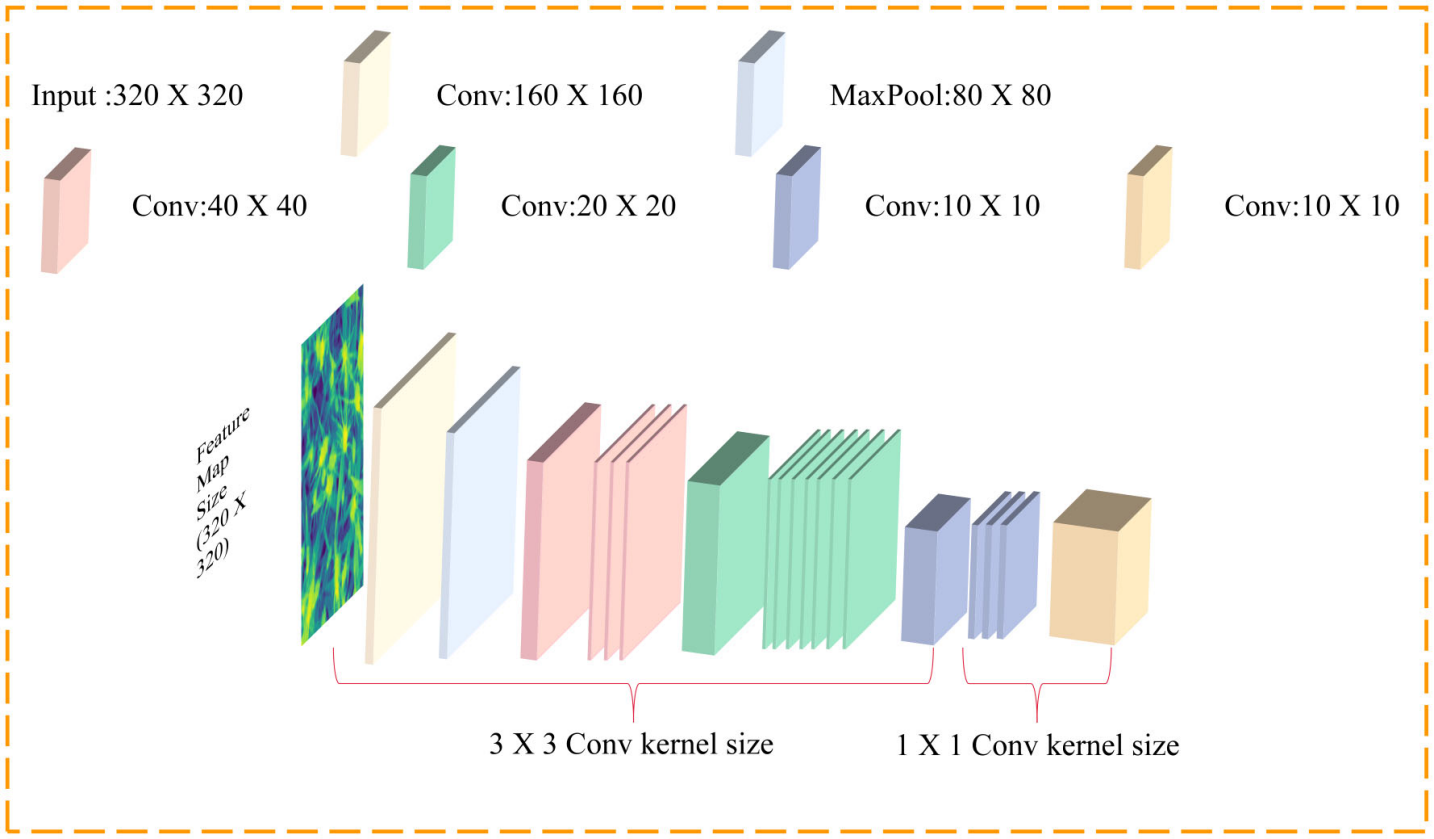
ShuffleNetV2 network architecture.

ShuffleNetV2 (Ma et al., 2018) is a lightweight neural network architecture designed specifically for mobile and embedded devices. It reduces computational and memory overhead while maintaining high accuracy by introducing techniques such as ShuffleUnit, grouped convolution, and channel shuffling. ShuffleUnit utilizes channel interleaving and lightweight convolution methods to promote information flow and feature mixing. Grouped convolution performs independent convolution operations on grouped input channels and then concatenates the results to reduce computational complexity and enhance information exchange between features. Channel rearrangements the channels of input feature maps into smaller groups, reducing parameters and computational overhead. The architecture of ShuffleNetV2 is illustrated in **Figure 4**.

#### 3.2.3 CARAFE

During the recognition of wheat ears, issues such as mutual shadowing between the ears of wheat and a poor ability to distinguish between the colors of the wheat and weeds can impair the extraction of features and significantly impact the detection accuracy. As a result, more details about edges, textures, and colors must be extracted. However, when using a conventional upsampling method (such as bilinears interpolation or transposed convolution, etc.) to map a low-resolution image with fewer pixels to the target high-resolution image, this step merely expands the size of the feature map rather than adding more specific details. Consequently, CARAFE (Wang et al., 2019) is introduced without significantly increasing the parameter to replace the upsampling method in the PANet. CARAFE is a lightweight, general-purpose upsampling operator that optimizes image detail and quality by feature reassembly and fusion, making the generated high-resolution feature maps more realistic and distinct.

**Figure 5:**
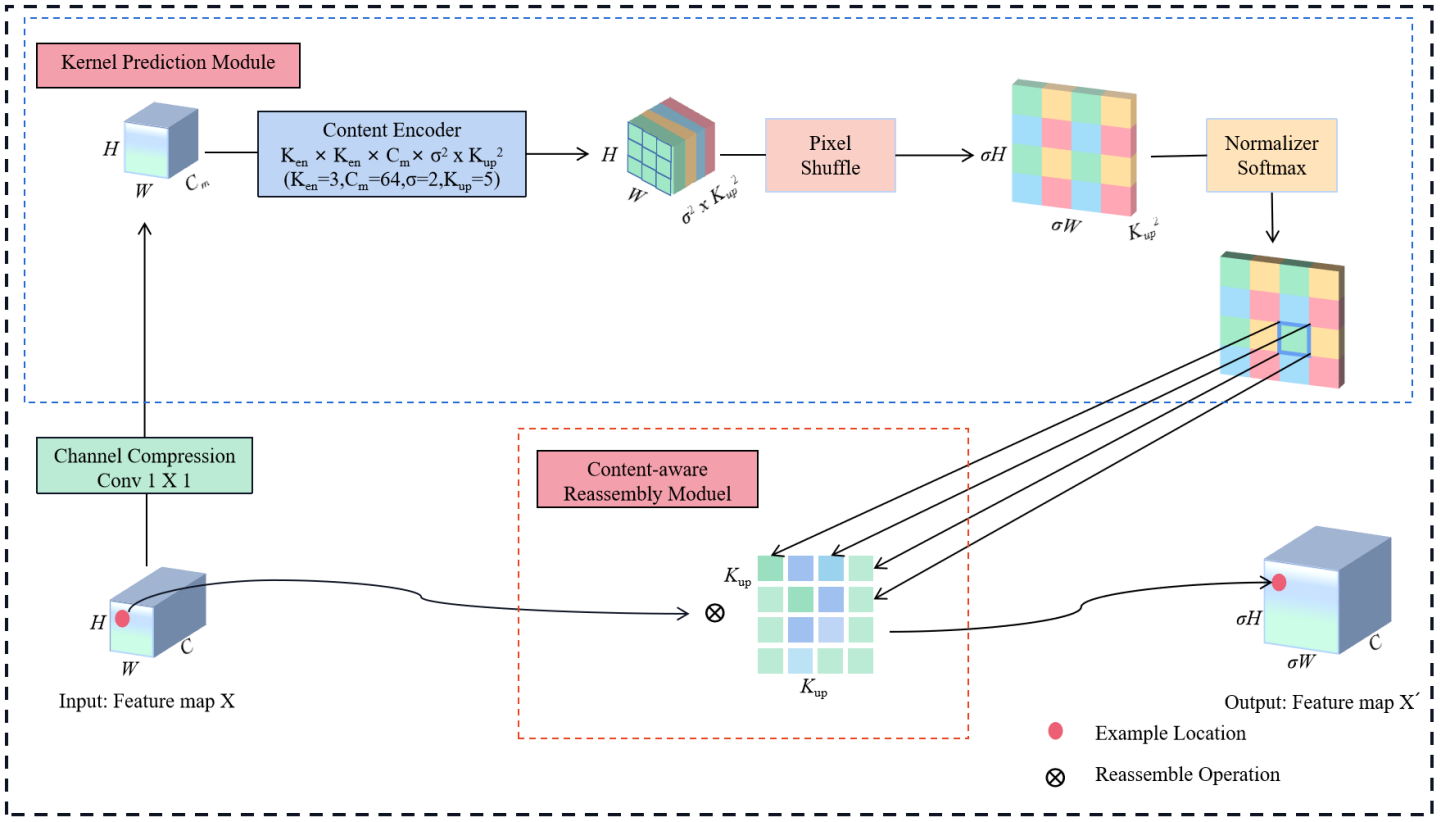
The structure of CARAFE. C represents the input channel of the feature map; H is the height of the image; W is the image width; C_m_ is the compressed channel; K_en_^2^ is the encoder size; σ is the upsampling ratio, and K_up_^2^ is the reassembly kernel size.

CARAFE comprises a kernel prediction module and a content-aware reassembly module. Through these two modules, it has the capacity to generate output feature maps X´ (σH × σW × C) from the input feature maps X (H × W × C). Specifically, the kernel prediction module utilizes the input feature map for predicting the reassembly kernel, while the content-aware reassembly module performs pointwise multiplication between the predicted reassembly kernel and the feature map obtained from conventional up-sampling completing the feature reassembly. The structural representation of CARAFE is illustrated in **Figure 5**.

#### 3.2.4 DyHead

**Figure 6:**
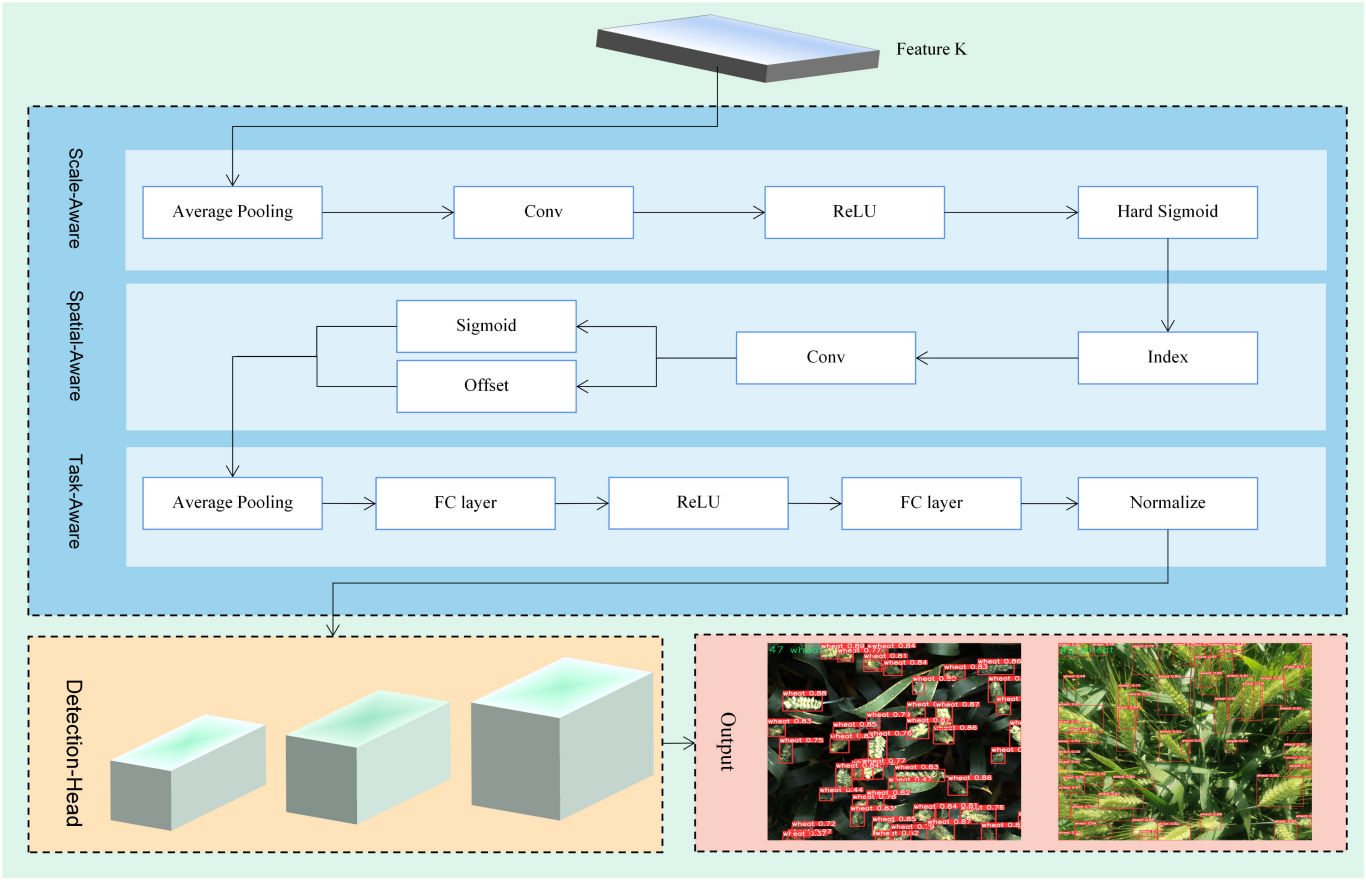
Workflow diagram of the detection head.

Wheat ears closest to the device seem larger than those farther away due to the perspective shifts of the camera in wheat ears detection. Furthermore, variations in shooting angles can lead to differences in the shape and relative locations of wheat ears, making it difficult for the model to interpret and use spatial information in the photos correctly. At the same time, this study hopes to express wheat ears in a diversified manner by employing multi-level representation methods such as feature points or detection boxes, which is intended to enable the algorithm to showcase optimal performance in specific tasks. In order to address these challenges and enhance the robustness of the wheat ears recognition model, DyHead is introduced as part of the head layer of the model. DyHead (Dai et al., 2021) is a dynamic object detection head based on attention mechanisms, which considers improvements in detection performance from three dimensions: scale perception, spatial perception, and task perception. The corresponding multiple attention mechanisms are effectively combined and integrated into a unified framework, addressing the feature layer of scale perception, spatial location for spatial perception, and the output channel for task perception. This significantly enhances the representation of the target detection head and successfully resolves issues related to perspective changes, spatial transformations, and diverse representations. The adoption of the dynamic target detection head enables the model to better adapt to wheat targets in complex scenes. The workflow diagram of the detection head is shown in **Figure 6**.

#### 3.2.5 Model optimization

**Figure 7:**
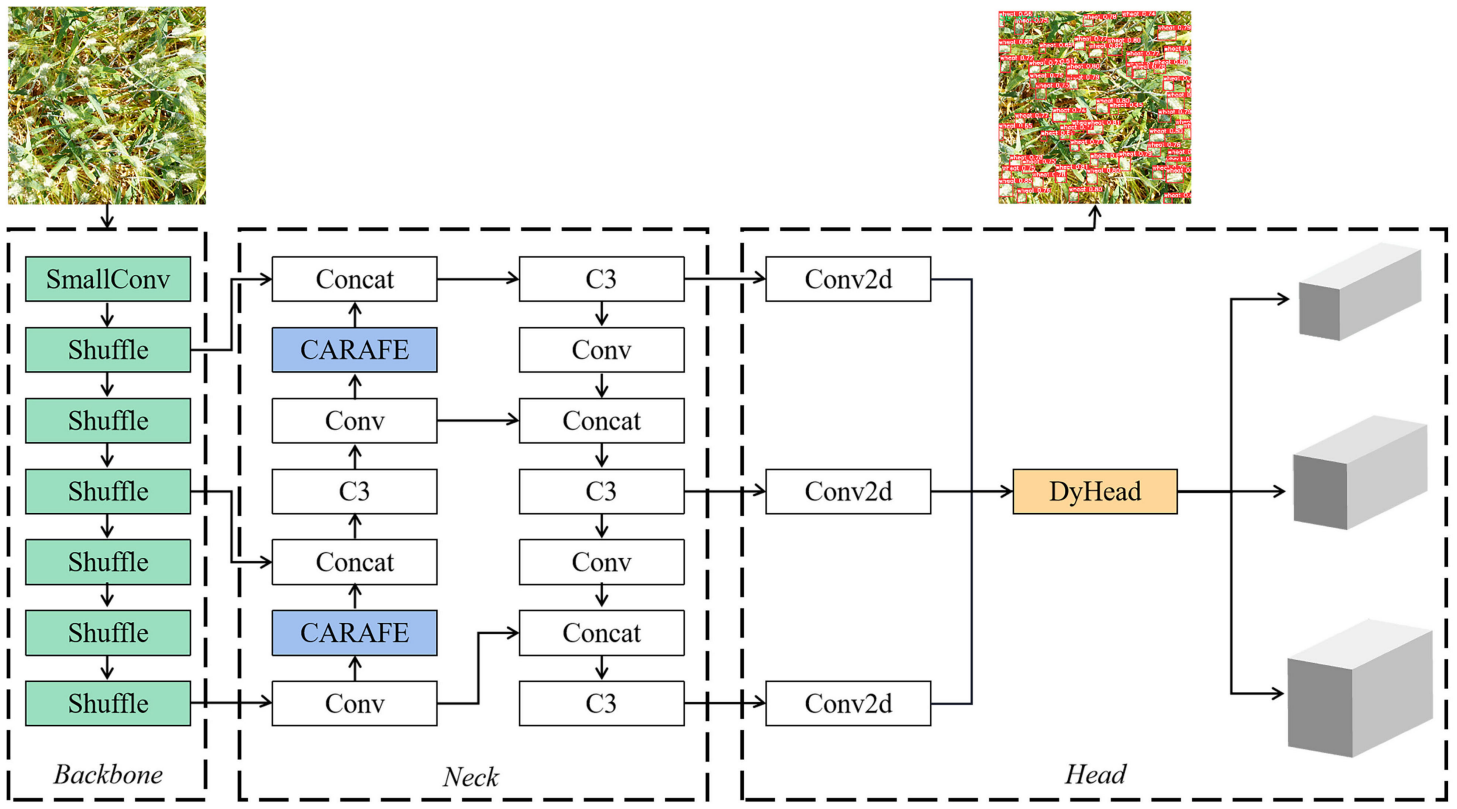
The network structure of S-YOLOv5s.

This paper proposes an improved model named S-YOLOv5s, which organically combines the ShuffleNetV2, CARAFE, and DyHead methods. The model structure is illustrated in **Figure 7**, successfully reducing the computational cost and model parameters of the YOLOv5s while enhancing its compatibility with less efficient devices. Among these, the lightweight ShuffleNetV2 network can drastically reduce the model parameters and computational complexity by replacing the backbone network CSPDarknet of YOLOv5s. Meanwhile, the lightweight generalized upsampling algorithm CARAFE is employed to replace the traditional upsampling method in PANet to improve the model detection accuracy of the lightweight network. This substitution results in high-quality feature mappings using a limited number of additional parameters and computational effort. Then, the model incorporates a DyHead attention-based detection head, which introduces relevant attention to the three dimensions of scale, space, and task. It also adjusts the weights of objects at different scales and locations within the space of the model, consolidating the three dimensions of scalability, spatiality, and task into a single dynamic detection head, effectively enhancing model accuracy.

### 3.3 Model performance evaluation

The performance of the wheat ears detection algorithm is evaluated by mAP, coefficient of determination (R^2^), Floating-Point Operations (FLOPs), Frames per second (FPS). Mean Absolute Error (MAE) and Root Mean Square Error (RMSE). The Intersection over Union (IOU) value determines whether the detection box matches the wheat ears bounding box.

Average Precision (AP) is introduced to represent detection accuracy. AP is a metric that measures the accuracy of object detection predictions of the algorithm at different confidence degrees. The higher the AP value, the higher the accuracy of the network. mAP is the average of the AP values for all classifications. Speed is measured by FPS. The calculation for AP, mAP, and FPS as shown in Equations 1–3.


(1) $$AP=\int_{0}^{1}P(R)d(R)$$

(2) $$mAP=\frac{\sum_{k=1}^{n}{\left(AP\right)}_{k}}{n}$$

(3) $$FPS=\frac{frame}{time}$$

The accuracy of the model detection results was assessed using the coefficient of determination R^2^, the MAE, and the RMSE metrics. R^2^ is a statistical measure used to assess the fit performance of a model, with values ranging from 0 to 1. A value closer to 1 indicates a better-fit result. MAE and RMSE are indicators of the error between the predicted and actual values. Smaller MAE and RMSE values indicate smaller errors between predicted and actual values, indicating higher accuracy and performance of the model. The calculation for R^2^, MAE, and RMSE as shown in Equations 4–6.


(4) $$R^{2}=1-\frac{\sum_{j=1}^{m}{\left(S_{j}-\hat{S_{j}}\right)}^{2}}{\sum_{j=1}^{m}\sum_{j}{\left(S_{j}-{\bar{S}}_{j}\right)}^{2}}$$

(5) $$MAE=\frac{1}{m}\sum_{j=1}^{m}\left|S_{j}-\hat{S_{j}}\right|$$

(6) $$RMSE=\sqrt{\frac{\sum_{j=1}^{m}{\left(S_{j}-\hat{S_{j}}\right)}^{2}}{m}}$$

where *m* represents the number of wheat ears images, *S_j_* and *Sj* respectively denote the manually annotated number of wheat ears and the model-detected count of wheat ears in the *j-th* image, and *S_j_* represents the average wheat ear number.

## 4 Results

### 4.1 Experimental environment

A PyTorch 1.11.0 framework-based experimental environment was used to train a wheat image recognition model. The GPU was an NVIDIA GeForce RTX 3090 with 24 GB of video memory, and the CPU was an AMD EPYC7543 with 80 GB of RAM. CUDA 11.3 and CUDNN 8.2 were used to provide GPU acceleration.

**Figure 8:**
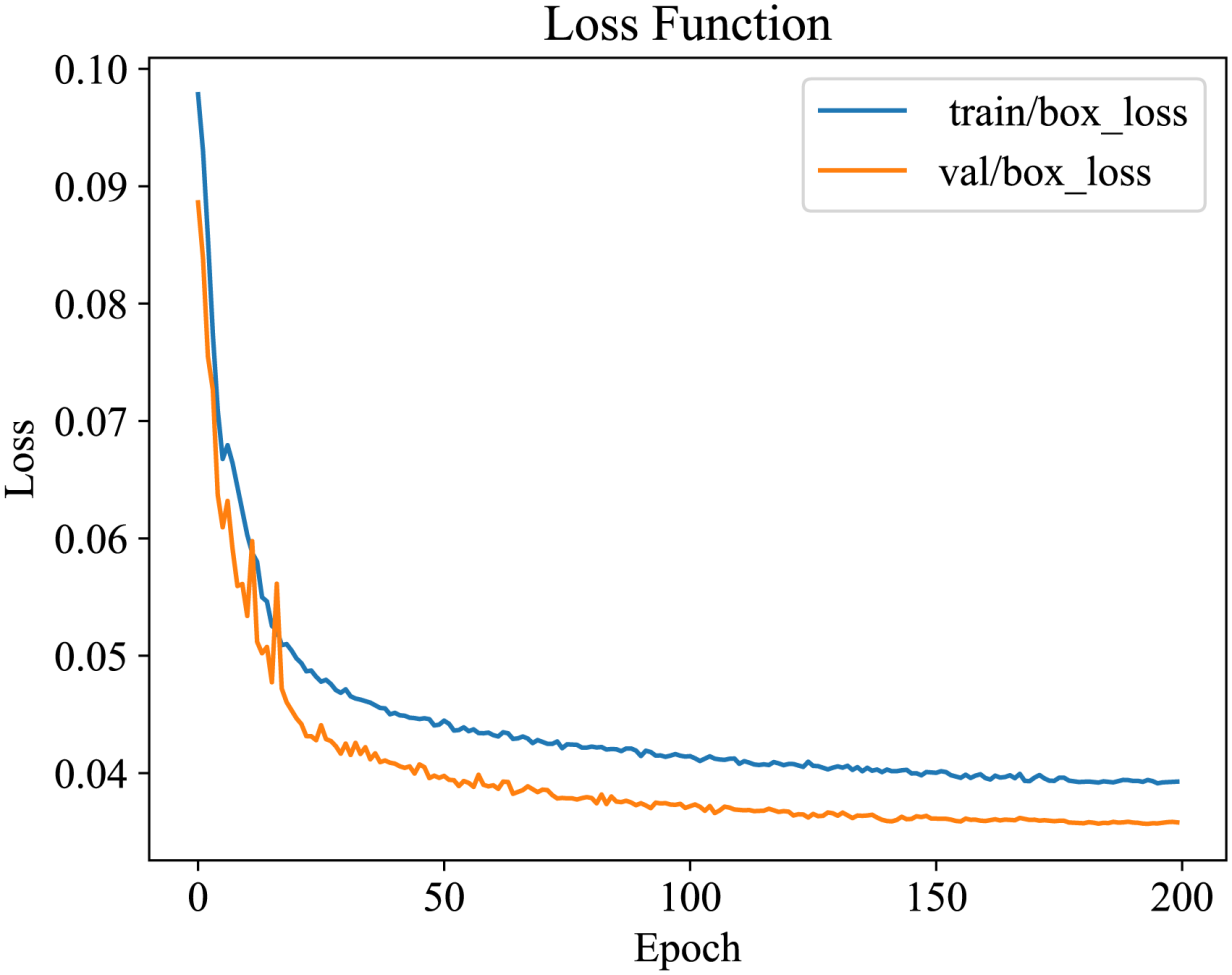
The loss function of the improved network S-YOLOv5s based on YOLOv5s.

Stochastic Gradient Descent (SGD) was employed as the optimizer throughout the model training, and the starting learning rate was set at 3E-2. The weight decay value was set at 0.937 to manage the complexity and prevent overfitting. Two hundred training epochs (iterative rounds) were conducted using the cosine annealing method for learning rate decay. In each epoch, a batch of 32 photos was utilized for training. By observing the convergence in the training process, the model started to show a convergence trend at close to 180 epochs. There was no overfitting, underfitting or gradient explosion problems in the whole training process, which indicates that the parameter settings used in the training process are appropriate, and the change of loss function is shown in **Figure 8**.

### 4.2 Ablation experiments

YOLOv5s is used as the baseline model, and improvement modules are gradually incorporated to assess their effectiveness through ablation experiments. Model performance is evaluated based on average precision, model weights, F1-Score, and computational complexity, as shown in **Table 3**.

**Table 3:** Ablation experiments.

ShufflenetV2	CARAFE	DyHead	Weight (MB)	FLOPs (10^9^)	Parameters (10^6^)	mAP@0.5 (%)	FPS
−	−	−	14.5	15.9	7.25	96.1	108
√	−	−	2.1	1.9	0.84	92.9	102
√	√	−	2.3	2.1	0.97	93.4	95
√	√	√	2.9	2.5	1.26	94.8	88

The experiment is divided into three stages to demonstrate the superiority of the S-YOLOv5s model. The ShuffleNetV2 lightweight network structure is incorporated in the first stage to replace the original backbone network. According to the findings, the weight of the model is 2.1 MB, which is 12.4 MB less than the original model weight, its computational volume is 1.9 * 10^9^, which is 14 * 10^9^ less, and its detection accuracy is 92.9%, which is 3.2% less. The results mentioned above demonstrate the effectiveness of the lightweight approach in the target detection task, showing that the model can still maintain high accuracy and strong detection performance. In the second stage, without significantly increasing the number of parameters or computational effort, the lightweight upsampling operator is further introduced to replace the upsampling operator of the neck module. As a result, the weight of the model, the computation, and the detection accuracy increased by 0.2 MB, 0.2 * 10^9^, and 0.5%, respectively. In the third stage, the DyHead was introduced to the head layer, and the weight of the model, the computational load, and the detection accuracy were increased by 0.6 MB, 0.4 * 10^9^, and 1.4%, respectively. The experimental results demonstrate that these optimization strategies successfully raise the performance of the model to a higher level while also contributing to its enhancement.

### 4.3 Comparison of different detection models

This study conducts comparative experiments with the top three state-of-the-art lightweight models in the current object detection field to demonstrate the effectiveness of the S-YOLOv5s model. Under the same conditions of training, validation, and testing sets, a comparison is made with the YOLOv6n, YOLOv7-tiny, and YOLOv8n models. The results are presented in **Table 4**.

**Table 4:** Comparison of detection results from different models based on the same dataset.

Model	Input (Resolution)	Weight (MB)	FLOPs (10^9^)	Parameters (10^6^)	mAP@0.5 (%)	FPS
YOLOv6n	640*640	9.97	11.34	4.3	93.4	102
YOLOv7-tiny	640*640	11.7	13.2	6.01	96.1	108
YOLOv8n	640*640	6.3	8.9	3.01	95.6	118
S-YOLOv5s	640*640	2.9	2.5	1.26	94.8	90

Comparative analysis shows that at an IOU value of 0.5, the mAP of the S-YOLOv5s model reaches 94.8%. The weight of model is merely 2.9 MB, with FLOPs at 2.5 * 10^9^, a parameter quantity of 1.26 * 10^6^, and an FPS of 88. Compared to YOLOv6n, YOLOv7-tiny, and YOLOv8n, S-YOLOv5s has reduced weights by 29%, 24.7%, and 46%, computational load by 22%, 18.9%, and 28%, and parameters by 29.3%, 20.9%, and 41.8%, respectively. In terms of mAP, it decreases by 1.3% and 0.8% compared to YOLOv7-tiny and YOLOv8n, while increasing by 1.4% compared to YOLOv6n. Although S-YOLOv5s has a lower FPS compared to YOLOv6n, YOLOv7-tiny, and YOLOv8n, it still exceeds 30 FPS, making it suitable for real-time detection tasks. The significantly reduced weights, parameter count, and computational load of S-YOLOv5s compared to other advanced lightweight detection models demonstrate the suitability of the lightweight model constructed in this paper for deployment on mobile devices.

### 4.4 Verifying model effects on different datasets

**Figure 9:**
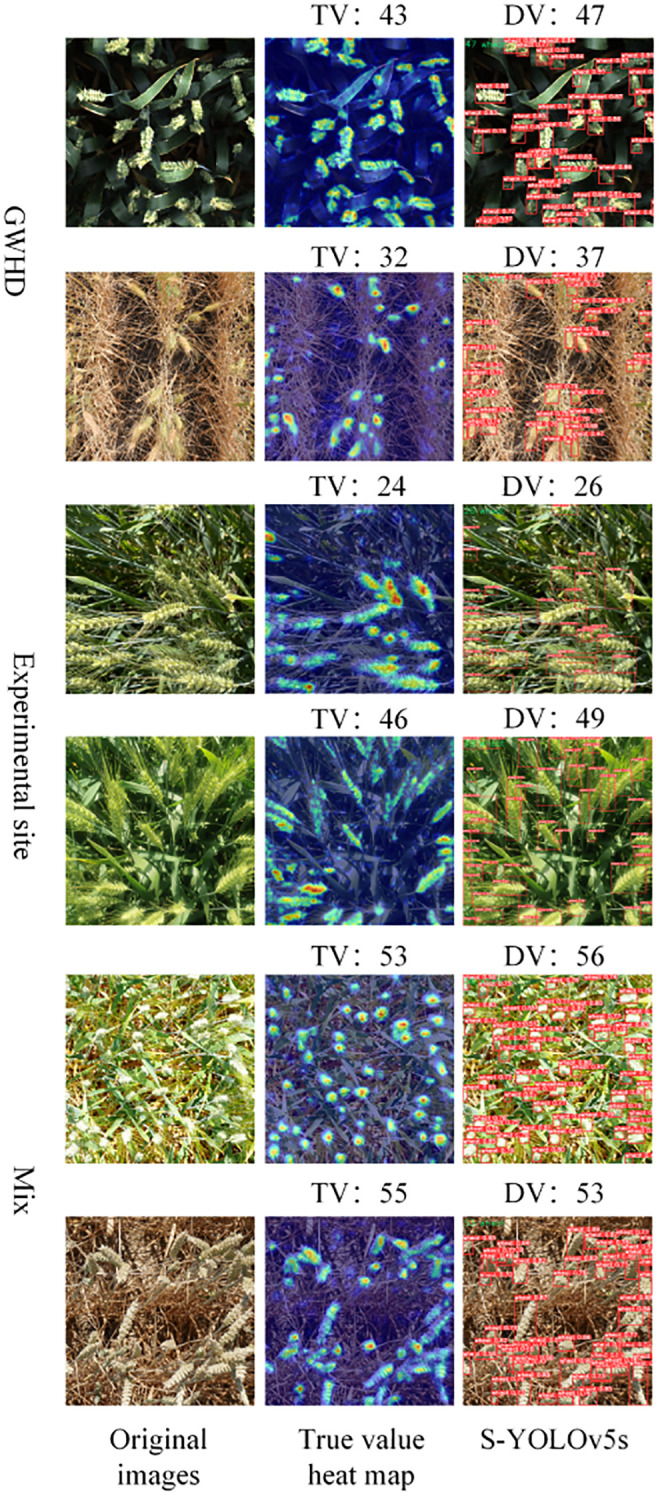
The detection counts of S-YOLOv5s were compared with the actual values in three different datasets. TV represents true value and DV represents detection value.

**Figure 10:**
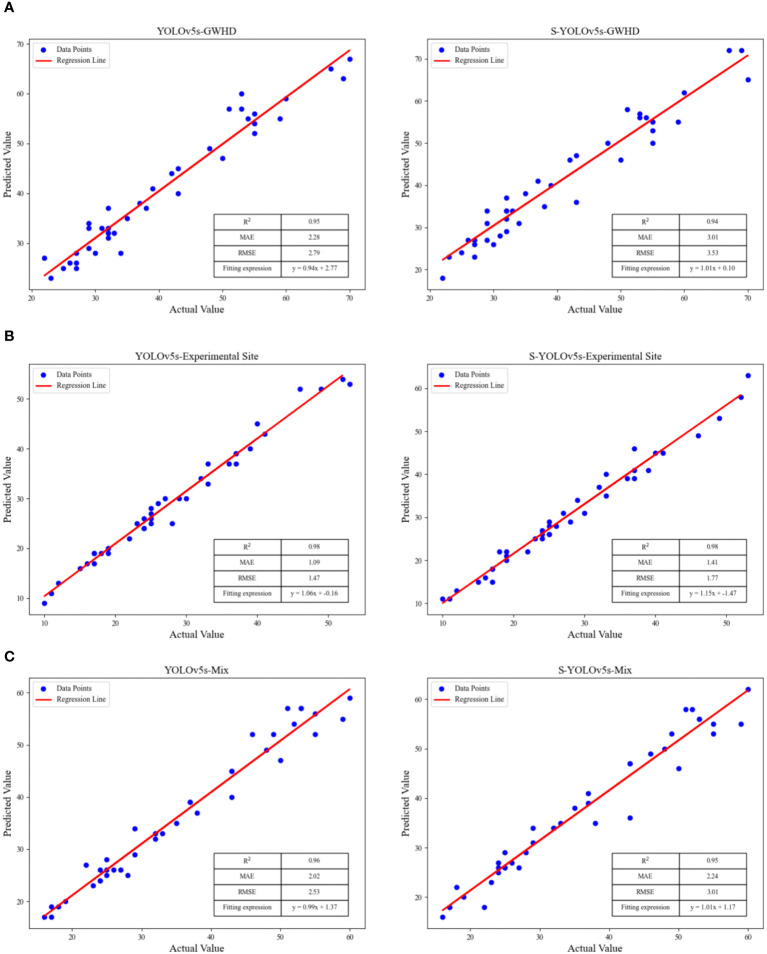
The linear fitting results of YOLOv5s and S-YOLOv5s for different wheat ears datasets, **(A)** the GWHD dataset, **(B)** the experimental site dataset, **(C)** the mixed dataset.

To validate the robustness, validity, and generalization ability of the model, 25 images from each of the two datasets were selected to form a mixed dataset (Mix). In this paper, the model results were validated in three datasets: the Global Wheat Head Detection (GWHD) dataset, the Experimental Site dataset and the Mixed Dataset. The test results of S-YOLOv5s are shown in **Figure 9**, and linear regression analysis was used to evaluate the prediction effects of the YOLOv5s, S-YOLOv5s models, as shown in **Figure 10**.

As shown in **Figure 8**, both methods demonstrate good testing accuracy across different datasets, and the three evaluation parameters of the constructed fitting curves are quite close. The S-YOLOv5s model exhibits better predictive performance on the experimental site dataset, with R^2^, MAE, and RMSE values of 0.98, 1.41, and 1.77, respectively. This is possibly due to the high clarity of the images in the experimental field dataset, allowing for more accurate extraction of wheat ears features and edge information. The S-YOLOv5s model shows slightly inferior predictive performance on the public dataset compared to the experimental site dataset, with R^2^, MAE, and RMSE values of 0.94, 3.01, and 3.53, respectively. The possible reason for this difference could be that the public dataset contains various wheat ears types with significant variations in shape, color, and texture, making it challenging for the model to capture subtle feature differences among them accurately. In addition, some images in the dataset may be blurry, which could lead to unclear edges and details of the target, negatively affecting the accuracy of predictions. Based on the testing results presented in this study, the S-YOLOv5s model achieves a balance between accuracy, reduced computational complexity, and model weights compared to YOLOv5s. Meanwhile, this research expands the dataset range, enhancing the detection performance of the model.

## 5 Discussion

This study introduces a wheat ears detection method called S-YOLOv5s. Compared to YOLOv5s, S-YOLOv5s experiences a 1.2% reduction in detection accuracy, primarily attributed to the use of a lightweight feature extraction network. While this network offers faster computation and fewer model parameters, it significantly reduces wheat ears feature extraction in complex scenes, potentially leading to instances of missed detections during the process. The size and diversity of the dataset are also crucial factors influencing the training results. Although the model achieved weight reduction by adopting a lightweight feature extraction network, the inference speed and detection speed of S-YOLOv5s did not show significant improvements, likely due to hardware limitations. Moreover, the choice of optimization techniques plays a crucial role in determining the inference speed of the model. Exploring optimization methods such as model quantization, pruning, and hardware acceleration can be considered to further enhance the efficiency of the model.

This study also compares the proposed algorithm against the detection results of other relatively lightweight YOLO structure-based wheat ears recognition studies, as shown in **Table 5**. Yan et al. (2022) proposed a lightweight self-attention wheat spike detector, LE-SPSANet, which utilized an asymmetric lightweight feature extraction network to reduce model parameters. They also employ the TanhExp activation function to reduce model training time and accelerate inference speed, resulting in a mAP of 94.4%. The model weight is 9 MB, with an FPS of 25. Rui and Yan (2022b) introduced a four-fold downsampling technique in the feature pyramid of YOLOv5 to increase the receptive field and enhance the detection capability for small objects. In addition, they incorporated a Convolutional Block Attention Module (CBAM) model into the neural network, which combines spatial attention and channel attention. This integration aimed to address the issue of gradient vanishing during the training process while simultaneously improving the feature extraction capability. The achieved mAP was 94.3%. Wu et al. (2023) optimized YOLOv7 by incorporating the Global Context Network (GCNet) and the Coordinated Attention (CA) mechanism in the backbone network to effectively utilize wheat characteristics. They introduced the Full-Dimensional Dynamic Convolution (ODConv) design into the network structure, which enhanced the interaction between dimensions and improved the performance of the detection model. The model weight is 40.4 MB, with a mAP of 96.2%.

**Table 5:** Comparing with the methods proposed in other wheat spike recognition studies.

Model	Weight (MB)	mAP (%)	FPS
LE-SPSANet	9.0	94.4	25
YOLOv5-CBAM	NA	94.3	NA
Improved-YOLOv7	40.4	96.2	14
S-YOLOv5s	2.9	94.8	90

Based on the comparative results, the proposed S-YOLOv5s demonstrates higher recognition rates than the LE-SPSANet and attention-based YOLOv5 detection methods. In terms of weight, the LE-SPSANet detection method surpasses S-YOLOv5s by 6.1 MB. Although the detection accuracy of S-YOLOv5s is 1.4% lower compared to the improved YOLOv7, it has significant advantages in weight and detection speed.

In summary, the lightweight wheat ears detection network S-YOLOv5s constructed in this study has improved model efficiency and adaptability to resource-constrained scenarios while maintaining high detection accuracy. In real-time detection scenarios, it can better cope with limited computational resources. The following steps of this research will focus on increasing the size and diversity of the dataset to improve the generalization and detection accuracy of the proposed S-YOLOv5s. The issue of missed detections in complex scenes, such as occlusions might be solved using more advanced feature extraction methods, like incorporating multi-scale feature fusion, to enhance the robustness and generalization ability of S-YOLOv5s. There is still room for optimization in the existing network structure, and other acceleration techniques can be applied to improve the inference and detection speed.

## 6 Conclusion

This study presents S-YOLOv5s, a lightweight wheat ears detection network based on a modified YOLOv5s architecture. By integrating the ShuffleNetV2 lightweight network to replace the backbone network CSPDarknet of YOLOv5s, the model significantly reduces parameters and computational costs while enhancing feature communication. The use of the lightweight CARAFE usampling operator in this model optimizes traditional upsampling in the PANet, enhancing edge information extraction. The model leverages DyHead, a dynamic target detection head based on an attention mechanism, to enhance feature fusion and detection performance.

The improved lightweight wheat ears detection network, S-YOLOv5s, achieves a mAP of 94.8%, slightly lower than the original YOLOv5s by 1.3%. The model weighs 2.9 MB, has 1.26 * 10^6^ parameters, and performs 2.5 * 109 FLOPs, constituting 20%, 17.3%, and 15.7% of the original YOLOv5s, respectively. The R^2^ for GWHD and the experimental site are 0.94 and 0.98, respectively. Compared to the original YOLOv5s, S-YOLOv5s only exhibits a slight decrease of 0.01 on the GWHD dataset, demonstrating that the lightweight wheat ears detection model, S-YOLOv5s, still possesses excellent detection performance. This study also compares S-YOLOv5s with three advanced lightweight object detection models, YOLOv6n, YOLOv7-tiny, and YOLOv8n. The results indicate that S-YOLOv5s excels in terms of model weight, parameter count, and computational load. Therefore, S-YOLOv5s is more easily deployable on memory-limited devices with low computational power, enabling mobile and real-time wheat spike recognition tasks without relying on expensive high-performance processing devices. Future research will introduce methods such as model quantization, pruning, hardware acceleration, and knowledge distillation to further optimize the model and enhance its deployment and detection capabilities.

## Data availability statement

The raw data supporting the conclusions of this article will be made available by the authors, without undue reservation.

## Author contributions

XS: Software, Writing – original draft, Investigation, Validation. CZha: Methodology, Writing – original draft, Supervision. KL: Investigation, Writing – review & editing, Validation. WM: Validation, Writing – review & editing. CZho: Investigation, Writing – review & editing. LY: Methodology, Writing – review & editing, Conceptualization, Data curation, Funding acquisition, Resources.
